# 

**Published:** 1918-10-19

**Authors:** 


### Gifts and Bequests.

The Great Northern Central Hospital has received from the trustees of the will of Lieutenant Alexander Wernher the sum of £500 from the amount bequeathed by him for division amongst charities. It also received from the staff of the Argentine Estates of Bovril, Ltd., a remittance of £39 for the maintenance of the Santa Elena Bed in the hospital's Military Annexe, Manor Gardens, and £200 from the directors of the London City and Midland Bank.

Mr. A. J. Atkinson, of Higher Broughton, has left £1,000 each to Dr. Stephenson's Orphan Home and the Manchester Royal Infirmary.

Miss Ada Bickersteth, of Hunton Bridge, Herts, amongst a number of legacies, left £500 each to the Middlesex Hospital for the Cancer Wards and the Meath Home for Epileptics.

The Eversfield Chest Hospital has received five hundred guineas from a "friend" to name a ward in the Eversfield Hospital for Consumption, St. Leonards on-Sea, in memory of his wife.

The Surrey Branch of the Red Cross Society has received £1,001, the proceeds of a cricket match at Kennington Oval on August Bank Holiday between England and the Dominions.

Mr. Ernest Crewdson, a chartered accountant, of Derby, left £1,000 to the Chartered Accountants' Benevolent Society, and £500 each to the Manchester and Salford Sick Poor Society and the Manchester Ear Institute.

Under the estate of Mr. Alfred Bax, F.S.A., a barrister of the Middle Temple, £1,000 is left to the Royal Hospital for Incurables.

The Manchester Royal Infirmary has received a donation of £1,000 from Mr. Frank Barlow to endow a bed in memory of his son, Lieut. Harold Barlow.

Mr. H. E. Acklom, of Bournemouth, has left £1,000 to the Royal Victoria and West Hants Hospital, and £300 each to the Royal National Sanatorium, Herbert Home, and Firs Home. He also left £500 to the London Jews' Society for the upkeep of a bed in the English Mission Hospital in Jerusalem.

Mr. Robert Gordon, of Christchurch, Hants, left £1,000 to Guy's Hospital, £500 each to King Edward's Hospital Fund and the Victoria Hospital for Children, Chelsea, £250 to the Dumfries and Galloway Infirmary, and £100 each to the Dorking Cottage Hospital and the Holmsdale Cottage Hospital, Kent.

Mr. Travers Duncan, of Belfast, has left £100 to the Royal Victoria Hospital.

Mr. Clement Stephenson, D.Sc., F.R.C.V.S., of Newcastle-on-Tyne, left £5,000 to the Armstrong College, Newcastle, for building and equipping laboratories, £1,000 to the Lying-in Hospital, £500 to the Fleming Memorial Hospital for Sick Children, and numerous legacies to other institutions, the residue of the estate to be divided in the same proportion as the legacies.

Dr. John Walker, of St. Leonards-on-Sea, left £100 to each of a number of charities, including the London City Mission and the Ashley Down Orphanage, Bristol.

A gift of 50,000 dollars (£10,000), the first instalment of a grant of 150,000 dollars, has been forwarded to the Scottish Women's Hospitals by the American Red Cross, Washington.

### Hospital and Institutional Results.

**Royal Free Hospital.**—The expenditure has increased from £23,966 in 1915 to £32,447 in 1917; the subscription list has fallen from £1,495 to £1,241 in the same period, and had it not been for a number of substantial legacies the hospital would have found itself in serious financial straits. We notice that the grant from the trustees of the late Mr. S. R. Zunz is put down as a legacy. Under the regulations of the Central Funds (see Red Book, page 8, par. 20), this amount should be classed as a donation.

**Wolverhampton and Staffordshire General Hospital.**—The report contains eighty-eight pages of rather heavy paper. The board of management meets every Tuesday at 11 a.m. to invite direct personal or written communications affecting the management of the institution. There is a total debit balance of £4,337 on maintenance account, £1,769 of which accrued last year.

**Glasgow Royal Infirmary.**—The nursing staff of the institution, past and present, have come out very creditably in the matter of honours and decorations. One nurse has been awarded the Military Medal, eight the Royal Red Cross (Second Class), and eleven have been mentioned in dispatches. There was an excess of ordinary expenditure over ordinary income in 1917 of £29,371, but legacies were received amounting to £23,388.

**Liverpool Royal Infirmary.**—This hospital is one of the very few which show in the annual report the exact cost of the nursing of the hospital under salaries, uniforms, and provisions; the amount thus spent in 1917 was £4,830 out of a total ordinary expenditure of £29,541 and in respect of 296 average occupied beds. The treasurer's account shows a deficit on the year's working of approximately £7,400.

**Royal Liverpool Country Hospital for Children.**—This comparatively recently founded institution at Heswall (this is the eighteenth annual report) continues to carry on its excellent work. The feature of the year was the decision of the committee to sink a well in the grounds to obtain water, and thus to save an appreciable part of the £270 that the year's supply now costs. It is not explained why the hospital was built where it would cost £270 a year for the water required, a charge which represents approximately £2 per occupied bed.

**Royal Alexandra Infirmary, Paisley.**—The expenditure in 1917 was £15,271, as against £11,729 in 1916, but there were good increases under all heads of income, and at the end of the year there was a deficiency of only £688.

**Orphan Working School and Alexandra Orphanage.**—Last year seventeen boys and girls were admitted whose fathers had fallen in the war. Although legacies in 1917 amounted to £2,520, the deficit for the year was £5,923, which sum has been met by a further loan from the bankers, who have now advanced £21,000. It is hoped that the institution will benefit by an appeal issued by the Lord Mayor on behalf of orphanages in and near London.

